# CD4+ T cell reconstitution, T cell activation, and memory T cell subset composition in blood and gut of HIV-negative and ART-suppressed HIV-positive patients: implications for HIV persistence in the gut

**DOI:** 10.1186/1758-2652-13-S3-O1

**Published:** 2010-11-04

**Authors:** S Yukl, E Sinclair, L Epling, Q Li, A Shergill, K McQuaid, L Duan, B Hare, H Lampiris, A Haase, D Havlir, J Wong

**Affiliations:** 1 San Francisco VA Medical Center, San Francisco, USA; 2 University of California San Francisco, San Francisco, USA; 3 San Francisco General Hospital, San Francisco, USA; 4 University of Minnesota, Minneapolis, USA

## Background

HIV DNA and RNA levels in the gut exceed those in the blood up to 10-fold. The ileum and rectum differ in HIV levels and responses to intensification, suggesting that mechanisms of persistence may vary between sites. HIV persistence could be impacted by T cell activation, absolute CD4 levels, or composition of memory T cell subsets.

## Methods

Mucosal biopsies were obtained from the ileums and rectums of eight HIV-negative controls and 10 ART-suppressed HIV-positive patients. Isolated cells were analyzed for T cell composition (CD3, CD4, CD8), activation (CD38, HLA-DR), and memory subpopulations (CD45RO, CCR7, CCR5, CD27) using flow cytometry. CD4 numbers were also measured by immunohistochemistry. In two patients, HIV DNA was measured in sorted subpopulations of memory CD4+ T cells.

## Results

CD4 reconstitution appears to reach normal levels in the rectum (mean CD4%: HIV positive 51.9%; HIV negative 48.7%), but not the ileum (HIV positive 29.6%; HIV negative 44.2%). Ileal CD4 numbers appear normal in the lymphoid aggregates, but not the lamina propria. In both the ileum and rectum, T cell activation remains higher than in HIV-negative controls (Table 1)

**Table 1:** Mean T cell activation, by site and HIV status

Mean activation	PBMC* HIV negative	PBMC HIV positive	Ileum HIV negative	Ileum HIV positive	Rectum HIV negative	Rectum HIV positive
CD38+HLA-DR+ as % of CD4+T	2.8	6.1	22.2	24.7	10.1	19.4
CD38+HLA-DR+ as % of CD8+T	7.8	15.3	13.2	24.0	17.7	28.3

* PBMC: peripheral blood mononuclear cells

In three HIV-positive patients, the ileum harboured more effector memory (EM) CD4+ T cells (mean 41.6% of CD4+ T cells) than the PBMC (15.4%), but similar numbers of transitional memory (TM) CD4+ T cells (ileum: 11.2%; PBMC: 14.0%). HIV DNA concentrations in ileal EM (1 copy/2100 cells) were 25-fold higher than in blood EM (1 copy/54,000 cells).

## Conclusions

T cell reconstitution and activation differ between gut sites. The higher concentration of HIV DNA in the gut is not due to increased TM cell%, but could reflect differences in the rate of infection of memory subtypes. The abnormal immune activation and lack of CD4 reconstitution in the ileum could reflect ongoing replication or chronic virus production.

